# Navigating ethical challenges in the development and translation of biomaterials research

**DOI:** 10.3389/fbioe.2022.949280

**Published:** 2022-09-20

**Authors:** Michael D. Hunckler, Aaron D. Levine

**Affiliations:** ^1^ Woodruff School of Mechanical Engineering, Georgia Institute of Technology, Atlanta, Georgia, United States; ^2^ Parker H. Petit Institute for Bioengineering and Bioscience, Georgia Institute of Technology, Atlanta, Georgia, United States; ^3^ School of Public Policy, Georgia Institute of Technology, Atlanta, Georgia, United States

**Keywords:** research ethics, biomaterials, animal research ethics, human subjects research ethics, ethics training, compliance and enforcement, scientific societies and professional associations

## Abstract

Biomaterials--from implanted iron teeth in the second century to intraocular lenses, artificial joints, and stents today--have long been used clinically. Today, biomaterials researchers and biomedical engineers are pushing beyond these inert synthetic alternatives and incorporating complex multifunctional materials to control biological interactions and direct physiological processes. These advances are leading to novel strategies for targeted drug delivery, drug screening, diagnostics and imaging, gene therapy, tissue regeneration, and cell transplantation. While the field has survived ethical transgressions in the past, the rapidly expanding scope of biomaterials science, combined with the accelerating clinical translation of this diverse field calls for urgent attention to the complex and challenging ethical dilemmas these advances pose. This perspective responds to this call, examining the intersection of research ethics -- the sets of rules, principles and norms guiding responsible scientific inquiry -- and ongoing advances in biomaterials. While acknowledging the inherent tensions between certain ethical norms and the pressures of the modern scientific and engineering enterprise, we argue that the biomaterials community needs to proactively address ethical issues in the field by, for example, updating or adding specificity to codes of ethics, modifying training programs to highlight the importance of ethical research practices, and partnering with funding agencies and journals to adopt policies prioritizing the ethical conduct of biomaterials research. Together these actions can strengthen and support biomaterials as its advances are increasingly commercialized and impacting the health care system.

## Introduction

Research ethics encompasses the set of rules, principles, and norms that guide responsible, morally acceptable, scientific research not just in biomaterials but across diverse fields of inquiry. Like almost all fields, biomaterials research has been caught up in research misconduct from time to time (Fanelli, 2009; Cyranoski, 2015). Minimizing these violations is important for the field if scientists and engineers are to maintain public trust. Rather than focusing on these issues, which are often driven by larger systematic considerations, this perspective focuses on research ethics questions driven specifically by the nature of ongoing advances in biomaterials research itself. These include biomaterials research using animal models, clinical research engaging human research subjects, and research using other emerging and contentious techniques. Our examples do not encompass every relevant research ethics principle, but rather illustrate those especially relevant to biomaterials. We hope this approach illuminates some of the tensions inherent in modern biomedical and bioengineering research and helps practicing biomaterials scientists and engineers navigate these challenges.

### Ethical considerations in preclinical animal research

Animals are indispensable to the development of biomaterials, especially in the context of clinical translation (Liguori et al., 2017), yet the use of animals raises ethical concerns for many stakeholders. Key ethical principles for animal research, articulated by Russell and Burch in 1959 (Russell and Burch, 1959), focus on replacing, reducing and refining animal use. Biomaterials scientists should draw on these principles—known as the 3Rs—in their experimental designs, utilize strategies (including those listed below) to comply with them when possible and be aware of biomaterials’ important role developing additional strategies that can help reduce or replace animal use in research over the longer term (Tannenbaum and Bennett, 2015).

*In Vitro Replacement of Animals:* The complex immune, mechanical, and cellular factors that animal models provide cannot be mimicked with *in vitro* assays when assessing host-biomaterial interactions. However, recent advances in tissue-/organ-on-a-chip technology, microfluidics, and bioprinting are being used to explore isolated physiological systems by mimicking highly complex microenvironments (Gibbons et al., 2012). Many of these biomaterial-based platform technologies provide enhanced spatial and temporal control compared to *in vivo* animal models and are, thus, positioned as potential alternatives to animal models, future cost-reducers in preclinical development, and valuable time-saving preclinical models (Huh et al., 2010). While cell monolayers have long been used for drug screening or toxicology (Davila et al., 1998), biomaterials have enabled multi-dimensional, physiologically similar construction of tissues that are more capable of predicting *in vivo* tissue functions and drug activities (Bhatia and Ingber, 2014; Shuler, 2017). Combining biomaterials and microfluidics has played a pivotal role in allowing precise manipulation of chemical gradients, fluid dynamics, three-dimensional tissue architecture, and cell behavior in *vitro* models (Kobel and Lutolf, 2011; Oliveira and Mano, 2014). Microfluidics are also being used for high-throughput screening of nanoparticles, further accelerating clinical translation and replacing animal models for arguably more effective and time-efficient models (Valencia et al., 2012). State of the art biofabrication and bioprinting are also being used for complex *in vitro* models of biomaterial-cell interactions (Bajaj et al., 2014; Seol et al., 2014).

*Consolidation of Preclinical Animal Testing:* In many circumstances, animal testing should not be replaced with *in vitro* tests; however, well-designed large animal studies can effectively consolidate *in vivo* testing requirements in order to reduce the number of animals used (Hampshire and Gilbert, 2019). The U.S. Food and Drug Administration (FDA) and the Center for Devices and Radiological Health (CDRH) have recently implemented guidance that promote replacement, reduction, and refinement in *vivo* biocompatibility testing with International Standard ISO 10993–1 (Biological evaluation of medical devices). As summarized (Hampshire and Gilbert, 2019), this guidance recognizes that the large animal model studies can provide robust biological data that can be leveraged to replace small animal models, especially in tests for systemic toxicity, chronic implantation, and *in vivo* thrombogenicity. By acknowledging potential improvements in efficiency of medical device testing, the FDA and CDRH have contributed to reduced animal burden and a reduction in economic resources for biocompatibility testing of biomaterials. Biomaterial researchers should continue to cooperate across academic, industry, and regulatory bodies to address the 3Rs when conducting *in vivo* biomaterial testing.

### Ethical considerations in clinical human Research

As biomaterials progress from preclinical research to clinical research, key ethical considerations for human subjects research must be heeded (Emanuel et al., 2000). These considerations derive from the three ethical principles (respect for persons, beneficence, and justice) outlined in the 1979 Belmont Report (National Commission for the Protection of Human Subjects of Biomedical and Behavioral Research, 1978). In this section, we discuss examples of how biomaterials research poses some unique challenges when adhering to these requirements.

*Voluntary Withdrawal:* Respect for human subjects enrolled in research studies requires that participation be voluntary, including the ability to withdraw from research at any time. However, biomaterials pose a considerable challenge to this stipulation because of their often permanent and integrative nature. Many new biomaterials are being engineered for maximal integration with the host tissue (Wang et al., 2004; Moroni and Elisseeff, 2008) or designed to promote endogenous repair and regeneration (Editorial, 2009; Wu et al., 2018; Christman, 2019), thus complicating or eliminating the ability for participants to withdraw. Other products are designed to elicit minimal host-biomaterial interaction but remain permanent. In deep brain stimulation (DBS), for example, which is used to treat a variety of neuropsychiatric conditions, regions of the brain require permanently implanted wire leads, optical fibers, or magnetic nanoparticles that are designed for the lifetime of the subject (Chen et al., 2015; Lozano et al., 2019). However, strategies such as designing the biomaterial to be retrievable, are being employed, especially within the context of cell transplantation. For example, retrievability is a key consideration in islet transplantation for diabetes (Weaver et al., 2018; Weaver et al., 2019), and encapsulation of the islets within a single retrievable device is performed to ensure rapid and complete removal in case of an emergency. In the realm of nanoparticles for imaging and therapeutics, current research is focusing on improving clearance kinetics of the potentially toxic heavy metals and fluorophores (Longmire et al., 2008). Researchers should support voluntary withdrawal whenever feasible and clearly indicate during the consent process when the experimental approach does not allow such withdrawal and why such a design is required.

*Research after Traumatic Injury:* Some biomaterial products pose unique ethical challenges for informed consent because the target population might comprise patients with recent traumatic events who require placement of irreversible biomaterials. For example, can a patient with traumatic compound bone fractures be afforded enough time and harbor the mental faculties to make a fully informed decision regarding the use of a novel biomaterial fixation device (Stevens, 2008)? As this example illustrates, severely injured trauma patients can seldom provide appropriate informed consent for clinical research, hindering the effectiveness of novel biomaterial development designed for trauma cases (Dutton et al., 2008; Miskimins et al., 2019). However, the FDA does provide narrow guidelines for exceptions to informed consent in emergency research under 21 CFR50.24 (Biros, 2003). In addition, in some cases, patients with traumatic injuries can be viewed as having diminished or limited autonomy and the decision for them to participate in research can be made through alternative means, such as proxy or deferred consent (Biros et al., 1995; Levine, 1995).

*Inclusion of Pediatric Population:* In many biomaterial applications, the pediatric population is at risk of being overlooked due to increased regulatory hurdles associated with conducting research on pediatric populations and technical challenges associated with designing growth-accommodating implants (Dunn, 2008; Williams and Guldberg, 2015). Multiple efforts are underway to address these issues, from decellularized xenograft tissues specifically for pediatric populations, to “living biomaterials” that alter their properties at multiple length and time scales, such as growth-accommodating implants that expand and grow over time (Patel and Fisher, 2008; Feins et al., 2017; Hofferberth et al., 2020). These new approaches provide an example of technical advances potentially promoting more just research by facilitating inclusion of pediatric populations in the development of biomaterials. In most cases, neither analogous regulatory nor technical challenges are as acute in geriatric populations.

*Cost-Effectiveness of Precision Biomaterials:* Technologies such as precision biomaterials elicit concerns of cost-effectiveness and accessibility. Personalized or “precision” medicine is emerging as a contributor to skyrocketing costs of medicine, with a predicted market size of $100 billion by 2025 (Collins and Varmus, 2015). Among these “precision” technologies is precision biomaterials, which are being engineered to address the limitations of a “one-size-fits-all” approach to medicine (Aguado et al., 2018). As biomaterials are designed with increasing sophistication (Place et al., 2009), however, the costs of such complex products threaten to widen disparities in access. Estimating these costs is challenging given heterogeneity among products under development and the nascent state of the field. Stem cell-based tissue engineered tracheas, where a small case study of transplants provided under compassionate use in the United Kingdom found costs ranged from approximately $174,000 to $740,000 provides one example (Culme-Seymour et al., 2016). Autologous chimeric antigen receptor T (CAR-T) cell therapies approved in the U.S. and other jurisdictions for the treatment of certain hematologic malignancies provides another. The first CAR-T cell therapy (Tisagenlecleucel) was approved in the U.S. in 2017 with a list price of $475,000 (Imbach et al., 2018) and more recently approved products have been marketed with list prices ranging from $373,000 to $465,000. However, not all technologies are growing costlier and more complex. Novel strategies are also aiming to simplify biomaterial products, instead using acellular, off-the-shelf biomaterials that unlock the body’s own power for organization and endogenous self-repair (Weber et al., 2013; Christman, 2019; Kirkton et al., 2019). Developing approaches that minimize cost, when possible, and ensuring that the population of research subjects is well-aligned with the potential patient population are important components of just and equitable distribution of these technologies.

### Emergent technologies carry ethical challenges

Biomaterial engineers are increasingly exposed to interdisciplinary research that cuts across a wide array of fields, including cell biology, drug development, and gene editing. In this section, we briefly review ethical challenges in some related fields that regularly intersect with biomaterial research and are, thus, important for the conduct of ethical biomaterials science and engineering.

*Embryonic and Fetal-Derived Tissue:* Biomaterials are frequently incorporated with ethically contentious materials, such as fetal tissue or human embryonic stem cells (hESCs), to aid in immunoprotection and immunoregulation, provide biological signals, and improve cellular retention in the body (Kraehenbuehl et al., 2011; Marquardt and Heilshorn, 2016; Vegas et al., 2016; Abdeen and Saha, 2017; Mitrousis et al., 2018). Fetal tissue and hESCs are highly controversial and elicit powerful debates and public engagement (Hyun, 2010). These debates have led to intermittent cuts and moratoriums in public funding - most notably, the Bush-era restrictions on federal funding for hESC research (Robertson, 2010) and Trump Administration restrictions on federal funding for fetal tissue research in the United States (Reardon, 2019). They have also led to the development of state-level research funding within the United States (Karmali et al., 2010; Alberta et al., 2015) and prompted individual scientists to seek these alternative, more stable, funding sources or shift to less ethically contentious materials, such as induced pluripotent stem cells (iPSCs) or umbilical cord blood stem cells (Levine, 2011a; 2011b). Indeed, iPSC technology has made tremendous progress in the past decade, and iPSCs are now widely used with biomaterials in disease modeling, drug discovery, tissue engineering, and organoid development (Shi et al., 2017).

*Gene Therapy:* The pursuit of efficient delivery of gene-editing systems, such as CRISPR technology, has exposed the biomaterials community to the world of gene-editing ethics. CRISPR offers the potential to eradicate multiple diseases through molecular surgery of the human genome, and biomaterials are being implemented to assist in delivering this gene-editing payload to appropriate target cells (Glass et al., 2018). In particular, nanoparticles, such as lipid nanoparticles and gold nanoparticles, have emerged as an innovative solution to encapsulate or tether the CRISPR DNA, mRNA, or ribonucleoprotein complexes to achieve more targeted gene therapy (Givens et al., 2018; Tenchov et al., 2021). Most notably, Moderna’s and BioNTech/Pfizer’s COVID-19 mRNA vaccines (while not gene therapy) employed these lipid nanoparticles to deliver the mRNA payload, demonstrating remarkable efficacy and unprecedented development speed in the fight against SARS-CoV-2 (Shin et al., 2020). In clinical gene editing applications, safety concerns include the risk of off-target effects and the possibility of heritable germline editing, in which a patient could pass genetic changes to his/her descendants (Brokowski and Adli, 2019).

### A path forward

Biomaterials scientists and engineers, especially those engaging in translational work, face competing pressures as they design and conduct their research. These challenges are present in the context of ethically justified animal research (where scientists must, for example, balance a desire to minimize the number of animals used with a desire to generate sound data and meet funder mandates), as well as clinical research using human subjects (where the decision to expose human subjects to unknown risks with, for example, a novel biomaterial, can be especially challenging). These competing pressures can also arise at the intersection of biomaterials and other contentious research tools or topics.

Ultimately, the extent to which biomedical research complies with or deviates from relevant ethical norms depends on both individual and group decisions as well as institutional and societal factors. We will never eliminate unethical behavior in science, but we can and should strive to minimize it. Accordingly, adopting strategies to encourage ethical research is an important task for the scientific and science policy communities. Fortunately, compliance with ethical norms can be encouraged through a variety of approaches. These include training, the use of institutional ethics committees, the adoption of voluntary guidelines, or rules, requirements, or restrictions adopted by journals and/or funding agencies. We briefly review these approaches below, focusing on examples relevant to biomaterials research.

*Research Ethics Training:* Driven in part by mandates from funding agencies, research ethics training is now a common approach to raise awareness of ethical issues in research and promote more ethical behavior (Eisen and Berry, 2002; Berry et al., 2016). Although training in the responsible conduct of research is widespread, the methods and goals of this training vary (Plemmons and Kalichman, 2013). Research ethics training can be effective, but concerns have been raised that it is sometimes treated more like a compliance activity than an opportunity to reflect on and support ethical research practices (Anderson, 2016). Furthermore, research ethics training developed in response to broad funder mandates often focuses on general principles and is not tailored to the concerns of specific fields, such as biomaterials. Individual institutions and programs can develop and require more customized training on ethical issues relevant to specific fields and biomedical engineering and other programs with significant biomaterials-related content should consider adopting this approach.

*Institutional Ethics Committees:* Institutional ethics committees also play an important role encouraging compliance with ethical norms. Although the specific committees vary by country and research topic, the U.S. context where research using human subjects must typically be approved by an Institutional Review Board (IRB) and research using most non-human animals typically requires approval by an Institutional Animal Care and Use Committee (IACUC) are illustrative. IRBs review research protocols with the ethical principles for human subjects research in mind and can help ensure research protocols use appropriate informed consent procedures, strive to balance benefits and risks of the research, and select subjects justly. Similarly, IACUCs help ensure animal research proceeds in compliance with key ethical norms. Biomaterials scientists and engineers should, of course, comply with these committee’s rules. They should remember, however, that some key issues fall outside the scope of these committees and that they also miss some concerns (Heimer and Petty, 2010; Steensma and Kantarjian, 2014). Thus, compliance should be viewed as a minimum bar that scientists should exceed as they strive to ensure their research is designed and conducted ethically.

*Scientific Societies:* Compliance with ethical norms can also be encouraged or enforced by scientific societies, which are often well positioned to provide field-specific guidance. We enumerate six ethics-related activities (see Table 1) that various societies have undertaken to engage their members and the broader public in ethics-related discussions: 1) publicly available Code of Ethics, 2) suggested voluntary guidelines, 3) membership ethics pledge, 4) standing ethics committee, 5) ethics session at annual conference, and 6) accessible online archive of published ethics and policy documents (e.g., patient advisories, published commentaries, etc.). The International Society for Stem Cell Research (ISSCR) and International Society for Cell & Gene Therapy (ISCT), for example, have taken an active role articulating ethical concerns surrounding the direct-to-consumer marketing and provision of unproven cell-based therapies (Hyun et al., 2008; Dominici et al., 2015). These societies require members to agree to follow their specific guidelines as a condition of membership and reserve the right to terminate the membership of individuals who do not comply. Other academic societies have adopted broader Codes of Conduct to encourage ethical and appropriate conduct by their members, both at society events (such as annual conferences) and more broadly in their research and professional activities. These codes have an important signaling role, raising awareness of ethical considerations and highlighting the importance of ethical behavior to the broader academic community. Engagement of members with relevant ethical concerns can also be achieved at annual meetings. For example, the Society for Biomaterials (SFB) hosted a panel discussion on biomaterial ethics at its 2018 annual meeting, and the ISSCR and ISCT routinely host sessions devoted to ethical considerations at their meetings.

**TABLE 1:** Strategies for scientific societies to promote ethical research practices.

Strategy	Description
Member Code of Ethics	Develop and publish a set of expectations for members to follow. These could cover scientific conduct broadly or be more narrowly tailored toward society events (e.g. appropriate behavior at an annual meeting)
Voluntary Guidelines	Develop and publish voluntary guidelines for both members and other stakeholders (e.g., patients, clinicians, industry, etc.) articulating norms for ethical behavior across all levels of biomaterial research (from basic science to clinical trials)
Membership Pledge	Require members to pledge compliance with code of ethics or other member guidelines. Refuse new membership or cancel current membership for non-compliance
Standing Ethics Committee	Create a standing ethics committee consisting of members (and potentially non-members) to advise society leadership and members on emerging ethical issues, and publicly comment on policy, regulatory, or ethical issues within the biomaterial field
Ethics Activities at Annual Meetings	Dedicate time at society meetings to raise awareness and promote discussion of ethical issues by, for example, inviting ethics speakers to present, organizing ethics-focused sessions and panel discussions, offering pre-/post-meeting workshops, etc.
Accessible Online Archive	Maintain a publicly available and accessible archive of ethics-related guidelines, legal commentary, advisories, and statements from other scientific societies adjacent to biomaterials with the potential for cross-cutting emergent ethical issues, and ethics activities to provide a resource to members and non-members

*Journals and Funding Agencies:* In addition, both funding agencies and journals can promote ethical research through influencing what research is conducted and disseminated. The influence of funding policies have been well documented for human embryonic stem cell research (Levine, 2008; Alberta et al., 2015) and similar effects may be seen for other areas where funding restrictions have been enacted, such as fetal tissue and human-animal chimera research (Reardon, 2016; Reardon, 2019). Journals can also influence the research enterprise by determining if, how, and under what conditions research can be disseminated. Journals can, for example, refuse to publish potentially problematic articles or refer them to other authorities for review, as has been the case for some high profile examples of dual-use research (Frankel, 2012). More common and arguably more important are the norms that journals enforce through submission or publication requirements. Journal requirements to post genomic sequence data to public databases, publicly archive research data, or report only on pre-registered clinical trial outcome measures, for example, can facilitate follow-up research, promote reproducibility and reduce bias in research (Vines et al., 2013; Warren, 2018).

## Discussion

Modern biomaterials research offers substantial promise to improve human health, yet the expanding scope of biomaterials research and the increased clinical translation of diverse biomaterials calls for attention to the ethical questions these advances pose. The field could, for instance, benefit from relevant academic societies (e.g., Society for Biomaterials, TERMIS, BMES, etc.) updating and adding specificity to their Codes of Conduct to more fully address biomaterials-specific concerns. Such codes could highlight key ethical considerations relevant to preclinical and clinical biomaterials research and clarify the expectations for ethical conduct by biomaterials researchers. The field could also prioritize improved ethics education within biomaterials training programs. Examples for developing ethics pedagogy for stem cell scientists already exist (Master et al., 2016), and could be used as a framework for developing biomaterials ethics courses, seminars, or workshops. These educational events could be organized at a variety of levels, such as individual biomedical engineering clubs, departmental efforts within institutions, funded graduate training programs, or scientific societies. Furthermore, additional panel discussions or focus sessions at annual conferences could engage researchers, policymakers, and industry leaders in the discussion of biomaterial ethics, especially as it pertains to interdisciplinary and emerging technologies. All of these are relatively low-cost steps that would raise awareness of the importance of ethics to the field and provide knowledge about best practices as well as resources and strategies to address potential research ethics issues as they arise.

Attention to research ethics is often reactive with policymakers or scientific societies responding to ethical lapses or scandals. The biomaterials community is not at this point. Rather the community has the opportunity to proactively engage with emerging and current ethical issues affecting the field. Given rapid progress toward clinical use of advanced biomaterials either alone or in combination with other novel therapeutic approaches, now is the time for the biomaterials community to engage proactively with these issues and prepare for the responsible translation of biomaterials research.

## Data Availability

The original contributions presented in the study are included in the article/supplementary material further inquiries can be directed to the corresponding author.

## References

[B1] Abdeen A. A. Saha K. (2017). Manufacturing cell therapies using engineered biomaterials. Trends Biotechnol. 35, 971–982. 10.1016/j.tibtech.2017.06.008 28711155 PMC5621598

[B2] Aguado B. A. Grim J. C. Rosales A. M. Watson-Capps J. J. Anseth K. S. (2018). Engineering precision biomaterials for personalized medicine. Sci. Transl. Med. 10, eaam8645. 10.1126/scitranslmed.aam8645 29343626 PMC6079507

[B3] Alberta H. B. Cheng A. Jackson E. L. Pjecha M. Levine A. D. (2015). Assessing state stem cell programs in the United States: How has state funding affected publication trends? Cell Stem Cell 16, 115–118. 10.1016/j.stem.2015.01.007 25658368

[B4] Anderson M. A. (2016). Pedagogical support for responsible conduct of research training. Hastings Cent. Rep. 46, 18–25. 10.1002/hast.533 26786037

[B5] Bajaj P. Schweller R. M. Khademhosseini A. West J. L. Bashir R. (2014). 3D biofabrication strategies for tissue engineering and regenerative medicine. Annu. Rev. Biomed. Eng. 16, 247–276. 10.1146/annurev-bioeng-071813-105155 24905875 PMC4131759

[B6] Berry R. M. Levine A. D. Kirkman R. Blake L. P. Drake M. (2016). Navigating bioethical waters: Two pilot projects in problem-based learning for future bioscience and Biotechnology professionals. Sci. Eng. Ethics 22, 1649–1667. 10.1007/s11948-015-9725-2 26563215

[B7] Bhatia S. N. Ingber D. E. (2014). Microfluidic organs-on-chips. Nat. Biotechnol. 32, 760–772. 10.1038/nbt.2989 25093883

[B8] Biros M. H. Lewis R. J. Olson C. M. Runge J. W. Cummins R. O. Fost N. (1995). Informed consent in emergency research: Consensus statement from the coalition conference of acute resuscitation and critical care researchers. JAMA 273, 1283–1287. 10.1001/jama.1995.03520400053044 7715041

[B9] Biros M. H. (2003). Research without consent: Current status, 2003. Ann. Emerg. Med. 42, 550–564. 10.1067/S0196-0644(03)00490-6 14520326

[B10] Brokowski C. Adli M. (2019). CRISPR ethics: Moral considerations for applications of a powerful tool. J. Mol. Biol. 431, 88–101. 10.1016/j.jmb.2018.05.044 29885329 PMC6286228

[B11] Chen R. Romero G. Christiansen M. G. Mohr A. Anikeeva P. (2015). Wireless magnetothermal deep brain stimulation. Science 347, 1477–1480. 10.1126/science.1261821 25765068

[B12] Christman K. L. (2019). Biomaterials for tissue repair. Science 363, 340–341. 10.1126/science.aar2955 30679357 PMC6697375

[B13] Collins F. S. Varmus H. (2015). A new initiative on precision medicine. N. Engl. J. Med. 372, 793–795. 10.1056/NEJMp1500523 25635347 PMC5101938

[B14] Culme-Seymour E. J. Mason K. Vallejo-Torres L. Carvalho C. Partington L. Crowley C. (2016). Cost of stem cell-based tissue-engineered airway transplants in the United Kingdom: Case series. Tissue Eng. Part A 22, 208–213. 10.1089/ten.tea.2015.0283 26559535 PMC4779280

[B15] Cyranoski D. (2015). Artificial-windpipe surgeon committed misconduct. Nature 521, 406–407. 10.1038/nature.2015.17605 26017424

[B16] Davila J. C. Rodriguez R. J. Melchert R. B. Acosta D. (1998). Predictive value of *in vitro* model systems in toxicology. Annu. Rev. Pharmacol. Toxicol. 38, 63–96. 10.1146/annurev.pharmtox.38.1.63 9597149

[B17] Dominici M. Nichols K. Srivastava A. Weiss D. J. Eldridge P. Cuende N. (2015). Positioning a scientific community on unproven cellular therapies: The 2015 international society for cellular therapy perspective. Cytotherapy 17, 1663–1666. 10.1016/j.jcyt.2015.10.007 26589750

[B18] Dunn J. C. Y. (2008). Tissue engineering and regenerative science in pediatrics. Pediatr. Res. 63, 459–460. 10.1203/PDR.0b013e3181739fbe 18360308

[B19] Dutton R. Stansbury L. Hemlock B. Hess J. Scalea T. (2008). Impediments to obtaining informed consent for clinical research in trauma patients. J. Trauma 64, 1106–1112. 10.1097/TA.0b013e318165c15c 18404082

[B20] Editorial (2009). Boom time for biomaterials. Nat. Mat. 8, 439. 10.1038/nmat2451 19458635

[B21] Eisen A. Berry R. M. (2002). The absent professor: Why we don’t teach research ethics and what to do about it. Am. J. Bioeth. 2, 38–49. 10.1162/152651602320957556 12762924

[B22] Emanuel E. J. Wendler D. Grady C. (2000). What makes clinical research ethical? JAMA 283, 2701–2711. 10.1001/jama.283.20.2701 10819955

[B23] Fanelli D. (2009). How many scientists fabricate and falsify research? A systematic review and meta-analysis of survey data. PLOS ONE 4, e5738. 10.1371/journal.pone.0005738 19478950 PMC2685008

[B24] Feins E. N. Lee Y. O’Cearbhaill E. D. Vasilyev N. V. Shimada S. Friehs I. (2017). A growth-accommodating implant for paediatric applications. Nat. Biomed. Eng. 1, 818–825. 10.1038/s41551-017-0142-5 29900036 PMC5995571

[B25] Frankel M. S. (2012). Regulating the boundaries of dual-use research. Science 336, 1523–1525. 10.1126/science.1221285 22723408

[B26] Gibbons M. C. Foley M. A. Cardinal K. O. (2012). Thinking inside the box: Keeping tissue-engineered constructs *in vitro* for use as preclinical models. Tissue Eng. Part B Rev. 19, 14–30. 10.1089/ten.teb.2012.0305 22800715

[B27] Givens B. E. Naguib Y. W. Geary S. M. Devor E. J. Salem A. K. (2018). Nanoparticle-based delivery of CRISPR/Cas9 genome-editing therapeutics. AAPS J. 20, 108. 10.1208/s12248-018-0267-9 30306365 PMC6398936

[B28] Glass Z. Lee M. Li Y. Xu Q. (2018). Engineering the delivery system for CRISPR-based genome editing. Trends Biotechnol. 36, 173–185. 10.1016/j.tibtech.2017.11.006 29305085 PMC5801045

[B29] Hampshire V. A. Gilbert S. H. (2019). Refinement, reduction, and replacement (3R) strategies in preclinical testing of medical devices. Toxicol. Pathol. 47, 329–338. 10.1177/0192623318797289 30270765

[B30] Heimer C. A. Petty J. (2010). Bureaucratic ethics: IRBs and the legal regulation of human subjects research. Annu. Rev. Law Soc. Sci. 6, 601–626. 10.1146/annurev.lawsocsci.093008.131454

[B31] Hofferberth S. C. Saeed M. Y. Tomholt L. Fernandes M. C. Payne C. J. Price K. (2020). A geometrically adaptable heart valve replacement. Sci. Transl. Med. 12, eaay4006. 10.1126/scitranslmed.aay4006 32075944 PMC7425635

[B32] Huh D. Matthews B. D. Mammoto A. Montoya-Zavala M. Hsin H. Y. Ingber D. E. (2010). Reconstituting organ-level lung functions on a chip. Science 328, 1662–1668. 10.1126/science.1188302 20576885 PMC8335790

[B33] Hyun I. Lindvall O. Ährlund-Richter L. Cattaneo E. Cavazzana-Calvo M. Cossu G. (2008). New ISSCR guidelines underscore major principles for responsible translational stem cell research. Cell Stem Cell 3, 607–609. 10.1016/j.stem.2008.11.009 19041777

[B34] Hyun I. (2010). The bioethics of stem cell research and therapy. J. Clin. Invest. 120, 71–75. 10.1172/JCI40435 20051638 PMC2798696

[B35] Imbach K. J. Patel A. Levine A. D. (2018). Ethical considerations in the translation of CAR-T cell therapies. Cell Gene Ther. Insights 4, 295–307. 10.18609/cgti.2018.030

[B36] Karmali R. N. Jones N. M. Levine A. D. (2010). Tracking and assessing the rise of state-funded stem cell research. Nat. Biotechnol. 28, 1246–1248. 10.1038/nbt1210-1246 21139604

[B37] Kirkton R. D. Santiago-Maysonet M. Lawson J. H. Tente W. E. Dahl S. L. M. Niklason L. E. (2019). Bioengineered human acellular vessels recellularize and evolve into living blood vessels after human implantation. Sci. Transl. Med. 11, eaau6934. 10.1126/scitranslmed.aau6934 30918113 PMC7557107

[B38] Kobel S. Lutolf M. P. (2011). Biomaterials meet microfluidics: Building the next generation of artificial niches. Curr. Opin. Biotechnol. 22, 690–697. 10.1016/j.copbio.2011.07.001 21821410

[B39] Kraehenbuehl T. P. Langer R. Ferreira L. S. (2011). Three-dimensional biomaterials for the study of human pluripotent stem cells. Nat. Methods 8, 731–736. 10.1038/nmeth.1671 21878920

[B40] Levine A. D. (2011a). Access to human embryonic stem cell lines. Nat. Biotechnol. 29, 1079–1081. 10.1038/nbt.2029 22158357

[B41] Levine A. D. (2008). Identifying under- and overperforming countries in research related to human embryonic stem cells. Cell Stem Cell 2, 521–524. 10.1016/j.stem.2008.05.008 18522844

[B42] Levine A. D. (2011b). Policy uncertainty and the conduct of stem cell research. Cell Stem Cell 8, 132–135. 10.1016/j.stem.2011.01.002 21295270

[B43] Levine R. J. (1995). Research in emergency situations: The role of deferred consent. JAMA 273, 1300–1302. 10.1001/jama.1995.03520400070049 7715046

[B44] Liguori G. R. Jeronimus B. F. de Aquinas Liguori T. T. Moreira L. F. P. Harmsen M. C. (2017). <sup/>Ethical issues in the use of animal models for tissue engineering: Reflections on legal aspects, moral theory, three rs strategies, and harm–benefit analysis. Tissue Eng. Part C. Methods 23, 850–862. 10.1089/ten.tec.2017.0189 28756735

[B45] Longmire M. Choyke P. L. Kobayashi H. (2008). Clearance properties of nano-sized particles and molecules as imaging agents: Considerations and caveats. Nanomedicine 3, 703–717. 10.2217/17435889.3.5.703 18817471 PMC3407669

[B46] Lozano A. M. Lipsman N. Bergman H. Brown P. Chabardes S. Chang J. W. (2019). Deep brain stimulation: Current challenges and future directions. Nat. Rev. Neurol. 15, 148–160. 10.1038/s41582-018-0128-2 30683913 PMC6397644

[B47] Marquardt L. M. Heilshorn S. C. (2016). Design of injectable materials to improve stem cell transplantation. Curr. Stem Cell Rep. 2, 207–220. 10.1007/s40778-016-0058-0 28868235 PMC5576562

[B48] Master Z. McDonald M. Paciulli D. Longstaff H. (2016). A primer on ethics education for stem cell and biomedical scientists. Curr. Stem Cell Rep. 2, 336–348. 10.1007/s40778-016-0064-2

[B49] Miskimins R. Pati S. Schreiber M. (2019). Barriers to clinical research in trauma. Transfusion 59, 846–853. 10.1111/trf.15097 30585332

[B50] Mitrousis N. Fokina A. Shoichet M. S. (2018). Biomaterials for cell transplantation. Nat. Rev. Mat. 3, 441–456. 10.1038/s41578-018-0057-0

[B51] Moroni L. Elisseeff J. H. (2008). Biomaterials engineered for integration. Mat. TodayKidlingt. 11, 44–51. 10.1016/S1369-7021(08)70089-0

[B52] National Commission for the Protection of Human Subjects of Biomedical and Behavioral Research (1978). The Belmont report: Ethical principles and guidelines for the protection of human subjects of research. Washington, D.C: DHEW Publication. (OS) 78-0012. 25951677

[B53] Oliveira M. B. Mano J. F. (2014). High-throughput screening for integrative biomaterials design: Exploring advances and new trends. Trends Biotechnol. 32, 627–636. 10.1016/j.tibtech.2014.09.009 25450043

[B54] Patel M. Fisher J. P. (2008). Biomaterial scaffolds in pediatric tissue engineering. Pediatr. Res. 63, 497–501. 10.1203/01.PDR.0b013e318165eb3e 18427294

[B55] Place E. S. Evans N. D. Stevens M. M. (2009). Complexity in biomaterials for tissue engineering. Nat. Mat. 8, 457–470. 10.1038/nmat2441 19458646

[B56] Plemmons D. K. Kalichman M. W. (2013). Reported goals of instructors of responsible conduct of research for teaching of skills. J. Empir. Res. Hum. Res. Ethics 8, 95–103. 10.1525/jer.2013.8.2.95 23651933 PMC3705638

[B57] Reardon S. (2019). Trump administration halts fetal-tissue research by government scientists. Nature 570, 148. 10.1038/d41586-019-01783-6 31186554

[B58] Reardon S. (2016). US agency to lift ban on funding human-animal hybrids. Nature 536, 135. 10.1038/nature.2016.20379 27510202

[B59] Robertson J. A. (2010). Embryo stem cell research: Ten years of controversy. J. Law Med. Ethics 38, 191–203. 10.1111/j.1748-720X.2010.00479.x 20579242

[B60] Russell W. M. S. Burch R. L. (1959). The principles of humane experimental technique. London: Methuen Publishing.

[B61] Seol Y.-J. Kang H.-W. Lee S. J. Atala A. Yoo J. J. (2014). Bioprinting technology and its applications. Eur. J. Cardiothorac. Surg. 46, 342–348. 10.1093/ejcts/ezu148 25061217

[B62] Shi Y. Inoue H. Wu J. C. Yamanaka S. (2017). Induced pluripotent stem cell technology: A decade of progress. Nat. Rev. Drug Discov. 16, 115–130. 10.1038/nrd.2016.245 27980341 PMC6416143

[B63] Shin M. D. Shukla S. Chung Y. H. Beiss V. Chan S. K. Ortega-Rivera O. A. (2020). COVID-19 vaccine development and a potential nanomaterial path forward. Nat. Nanotechnol. 15, 646–655. 10.1038/s41565-020-0737-y 32669664

[B64] Shuler M. L. (2017). Organ-body- and disease-on-a-chip systems. Lab. Chip 17, 2345–2346. 10.1039/C7LC90068F 28671705

[B65] Steensma D. P. Kantarjian H. M. (2014). Impact of cancer research bureaucracy on innovation, costs, and patient care. J. Clin. Oncol. 32, 376–378. 10.1200/JCO.2013.54.2548 24395852

[B66] Stevens M. M. (2008). Biomaterials for bone tissue engineering. Mat. TodayKidlingt. 11, 18–25. 10.1016/S1369-7021(08)70086-5

[B67] Tannenbaum J. Bennett B. T. (2015). Russell and burch’s 3Rs then and now: The need for clarity in definition and purpose. J. Am. Assoc. Lab. Anim. Sci. 54, 120–132. 25836957 PMC4382615

[B68] Tenchov R. Bird R. Curtze A. E. Zhou Q. (2021). Lipid nanoparticles—from liposomes to mRNA vaccine delivery, a landscape of research diversity and advancement. ACS Nano 15, 16982–17015. 10.1021/acsnano.1c04996 34181394

[B69] Valencia P. M. Farokhzad O. C. Karnik R. Langer R. (2012). Microfluidic technologies for accelerating the clinical translation of nanoparticles. Nat. Nanotechnol. 7, 623–629. 10.1038/nnano.2012.168 23042546 PMC3654404

[B70] Vegas A. J. Veiseh O. Gürtler M. Millman J. R. Pagliuca F. W. Bader A. R. (2016). Long-term glycemic control using polymer-encapsulated human stem cell-derived beta cells in immune-competent mice. Nat. Med. 22, 306–311. 10.1038/nm.4030 26808346 PMC4825868

[B71] Vines T. H. Andrew R. L. Bock D. G. Franklin M. T. Gilbert K. J. Kane N. C. (2013). Mandated data archiving greatly improves access to research data. FASEB J. 27, 1304–1308. 10.1096/fj.12-218164 23288929

[B72] Wang D.-A. Williams C. G. Yang F. Elisseeff J. H. (2004). Enhancing the tissue-biomaterial interface: Tissue-initiated integration of biomaterials. Adv. Funct. Mat. 14, 1152–1159. 10.1002/adfm.200305018

[B73] Warren M. (2018). First analysis of ‘pre-registered’ studies shows sharp rise in null findings. Nature. 10.1038/d41586-018-07118-1

[B74] Weaver J. D. Headen D. M. Coronel M. M. Hunckler M. D. Shirwan H. García A. J. (2019). Synthetic poly(ethylene glycol)-based microfluidic islet encapsulation reduces graft volume for delivery to highly vascularized and retrievable transplant site. Am. J. Transpl. 19, 1315–1327. 10.1111/ajt.15168 PMC6487074 30378751

[B75] Weaver J. D. Headen D. M. Hunckler M. D. Coronel M. M. Stabler C. L. García A. J. (2018). Design of a vascularized synthetic poly(ethylene glycol) macroencapsulation device for islet transplantation. Biomaterials 172, 54–65. 10.1016/j.biomaterials.2018.04.047 29715595 PMC5967258

[B76] Weber B. Dijkman P. E. Scherman J. Sanders B. Emmert M. Y. Grünenfelder J. (2013). Off-the-shelf human decellularized tissue-engineered heart valves in a non-human primate model. Biomaterials 34, 7269–7280. 10.1016/j.biomaterials.2013.04.059 23810254

[B77] Williams C. Guldberg R. E. (2015). Tissue engineering for pediatric applications. Tissue Eng. Part A 22, 195–196. 10.1089/ten.tea.2015.0514 26573765

[B78] Wu R.-X. Xu X.-Y. Wang J. He X.-T. Sun H.-H. Chen F.-M. (2018). Biomaterials for endogenous regenerative medicine: Coaxing stem cell homing and beyond. Appl. Mat. Today 11, 144–165. 10.1016/j.apmt.2018.02.004

